# GDM-DTM: A Group Decision-Making-Enabled Dynamic Trust Management Method for Malicious Node Detection in Low-Altitude UAV Networks

**DOI:** 10.3390/s25133982

**Published:** 2025-06-26

**Authors:** Yabao Hu, Yulong Gan, Haoyu Wu, Cong Wang, Maode Ma, Cheng Xiong

**Affiliations:** 1 College of Artificial Intelligence, Tianjin University of Science and Technology, Tianjin 300457, China; huyabao@tust.edu.cn (Y.H.); 22101104@mail.tust.edu.cn (Y.G.); 22101217@mail.tust.edu.cn (H.W.); wangcongjcdd@tust.edu.cn (C.W.); 24836964@mail.tust.edu.cn (C.X.); 2 College of Engineering, Qatar University, Doha P.O. Box 2713, Qatar

**Keywords:** trust management, group decision-making, UAV network, low-altitude economy

## Abstract

As a core enabler of the emerging low-altitude economy, UAV networks face significant security risks during operation, including malicious node infiltration and data tampering. Existing trust management schemes suffer from deficiencies such as strong reliance on infrastructure, insufficient capability for multi-dimensional trust evaluation, and vulnerability to collusion attacks. To address these issues, this paper proposes a group decision-making (GDM)-enabled dynamic trust management method, termed GDM-DTM, for low-altitude UAV networks. GDM-DTM comprises four core parts: Subjective Consistency Evaluation, Objective Consistency Evaluation, Global Consistency Evaluation, and Self-Proof Consistency Evaluation. Furthermore, the method integrates a Dynamic Trust Adjustment Mechanism with multi-attribute trust computation, enabling efficient trust evaluation independent of ground infrastructure and thereby facilitating effective malicious UAV detection. The experimental results demonstrate that under identical conditions with a malicious node ratio of 30%, GDM-DTM achieves an accuracy of 85.04% and an F-score of 91.66%. Compared to the current state-of-the-art methods, this represents an improvement of 6.04 percentage points in accuracy and 3.71 percentage points in F-score.

## 1. Introduction

As a new economic form, the low-altitude economy relies on low-altitude airspace resources and aviation technology to create an industrial ecosystem encompassing various dimensions such as research and development, manufacturing, flight services, and comprehensive support [1]. Centered around unmanned aerial vehicle (UAV) systems as the primary carrier, and through the deep integration of low-altitude flight activities with ground infrastructure, related UAV technologies such as multi-UAV communication, intelligent relay selection and deployment, and collaborative air–ground network optimization have also witnessed rapid development [2]. As a result, the low-altitude economy is gradually evolving into a strategic emerging industry that is being actively promoted by major global economies.

However, with the exponential growth of UAV networks, the security threats faced by the system are becoming increasingly complex. Attacks such as malicious node infiltration, communication link hijacking, and false information dissemination not only degrade network performance and compromise communication security [3,4], but also risk triggering catastrophic consequences like UAV loss of control and crashes. Consequently, these threats pose severe risks to stable low-altitude economic system operation. In addition, when UAVs carry out missions, they often face scenarios without dependence on ground infrastructure. For example, when performing outdoor surveying tasks, the openness of the communication environment and the dynamic nature of the network topology make traditional security measures ineffective.

In this context, trust management mechanisms have emerged as critical solutions for ensuring UAV network security by continuously assessing node trustworthiness, detecting malicious activities, and mitigating attack impacts. However, existing mechanisms still encounter notable limitations. Primarily, the reliability of malicious node detection remains low in scenarios without support from ground infrastructure. Most conventional trust management systems are centralized, such as those based on guarantee mechanisms [5] or reputation systems [6], which inherently face risks associated with single points of failure. To mitigate centralization risks, decentralized approaches including consensus-based trust evaluation [7,8,9] and blockchain-based trust storage [10,11,12] have been proposed. Nonetheless, these decentralized solutions still depend on auxiliary ground infrastructure, restricting their effectiveness in fully infrastructure-independent scenarios. In contrast, our proposed GDM-DTM method enhances malicious node detection performance by introducing a group decision-making (GDM) framework that does not rely on ground infrastructure, thus overcoming these critical limitations.

Secondly, existing trust modeling approaches remain inadequate when addressing complex attack scenarios. Traditional trust evaluation models primarily rely on single-dimensional metrics, such as historical interaction frequency or link quality [13,14], which limits their ability to detect sophisticated, multi-dimensional coordinated attacks. In response, recent studies have explored more comprehensive models by incorporating historical behavior patterns, reputation metrics, and contextual information [15,16,17,18]. These approaches represent important progress in enhancing trust model expressiveness. However, they still suffer from key limitations, including poor generalization across diverse environments and insufficient adaptability to rapidly changing network topologies. Particularly in dynamic UAV networks, where node behavior and connections fluctuate frequently, existing models struggle to maintain consistent and accurate trust assessments. In contrast, our proposed GDM-DTM framework addresses these issues by integrating multi-attribute trust computation with a group decision-making mechanism, thereby enabling dynamic and robust trust evaluation even in highly variable and adversarial conditions.

Based on these challenges, we propose a consistency evaluation trust management method based on group decision-making, called GDM-DTM, in low-altitude economy UAV networks. The main contributions of this paper are as follows:

- GDM-Based Trust Management: We integrate group decision-making (GDM) into the UAV trust management method, termed GDM-DTM. GDM-DTM evaluates and aggregates trust values based on four consensus parts to make consensus-driven decisions, while trust management governs UAV trustworthiness. This approach has significantly enhanced malicious node detection capability in scenarios without ground infrastructure.
- Multi-dimensional Trust Modeling: A trust value calculation method based on multi-dimensional UAV attribute data is proposed to provide a more reliable trust computation approach.
- Proactive Trust Validation via Self-Proof Consistency: To counter collusion and false trust propagation, we introduce a self-proof consistency concept for UAV environments and propose self-proof consistency degree as a new metric to evaluate consensus levels. GDM-DTM transforms trust validation from passive estimation to proactive provision, thus more effectively identifying and adjusting discrepancies in opinions.
- Dynamic Threshold and Weight Adjustment: A dynamic threshold adjustment algorithm based on attribute weight distribution and node tolerance is introduced to enhance the system’s environmental adaptability in dynamic topologies and novel attack scenarios.
- We conduct detailed evaluation experiments on the effectiveness of GDM-DTM in trust evaluation and malicious node detection. The experimental results show that GDM-DTM not only outperforms traditional methods in terms of performance but also demonstrates stronger adaptability and robustness when dealing with complex attack scenarios.

## 2. Related Work

### 2.1. UAV Trust Management

Trust management has become one of the main approaches for UAV security defense [19]. Trust management achieves malicious node detection through trustworthiness evaluation, with its core being the construction of dynamic quantification models to resist network attacks, such as message tampering attacks and the spread of false information.

In centralized architectures, Liu et al. [20] proposed a dynamic reputation evaluation model based on behavioral indicators. By using cryptographic techniques, the UAV’s behavioral characteristics are mapped to multiple threshold levels, allowing dynamic management of the UAV’s reputation and enabling real-time trust management led by the ground control station. However, centralized trust management suffers from the risk of single points of failure. To address this issue, Zheng’s team [13] proposed a semi-centralized framework integrating blockchain and federated learning, which improves evaluation accuracy while optimizing system energy efficiency, but still relies on specific nodes, limiting its adaptability to dynamic environments.

In distributed architectures, Kundu et al. proposed a trust-based dynamic leader selection mechanism (TDLS-FANET) [21]. This approach evaluates both trust scores and physical fitness to form secure UAV clusters and elect reliable leaders. While the scheme enhances network robustness and efficiency, it is sensitive to local measurement accuracy and may incur computational overhead. Liang et al. proposed a distributed trust evaluation mechanism that constructs direct trust certificates through active validation of information collection and introduced high-reputation nodes as auxiliary trust sources [22]. However, the potential malicious contamination of the initial node set may affect the reliability of indirect trust evaluations. Zhang’s team proposed a reputation-based distributed trust management mechanism using the Raft-PoA consensus to manage node trust levels [23], but this approach faces significant computational resource overhead. Table 1 presents a functional comparison between the GDM-DTM scheme and other existing schemes.

In methods combining machine learning, Akram et al. proposed a dynamic trust management mechanism that integrated adversarial learning and type-2 fuzzy logic to dynamically adapt to the environment [24], but the model’s performance depends on the quality of the training data. Akram et al. proposed TMIoDT [25], a decentralized trust management system integrating digital twins, blockchain, and deep learning-based intrusion detection for IoDT. Their system ensures transparency and adaptive trust scoring under adversarial conditions, though its complexity and dependency on training data limit lightweight UAV applications.

Although the aforementioned research has made significant progress in trust model architecture, evaluation mechanisms, and defense technologies, existing solutions still struggle to effectively meet the detection needs in environments that lack ground infrastructure, especially in areas such as collusion attack suppression, complex attack pattern recognition, and ensuring the credibility of group decisions. Therefore, there is an urgent need for a dynamic trust management method that integrates multi-dimensional trust evaluation and group consensus optimization to enhance system robustness and adaptability in complex attack scenarios.

### 2.2. Group Decision-Making

In group decision-making, the Consensus Reaching Process (CRP) guides decision-makers to iteratively adjust their strategies and modify opinion deviations until the group consensus level meets a predefined threshold. Regarding the modeling of bounded confidence and its relationship with CRP, Zhang et al. constructed a personalized feedback mechanism based on bounded confidence learning [26]. This mechanism integrates opinion acceptability evaluation and dynamic opinion adjustment algorithms, enhancing the robustness of the group decision-making system. Building on this, Zhou et al. developed a decision-making model based on trust networks and ordinal consensus metrics [27], determining decision-maker weights by combining mediator centrality and trust in-degree. This method comprehensively considers both the intensity of trust propagation and the topological influence of decision-makers within the trust network. In large-scale complex decision-making scenarios, Shen et al. developed a hybrid opinion dynamics model based on dynamic social networks [17]. By clustering experts and dynamically adjusting network structures, this model effectively improves the representation of the diversity and complexity of real decision-making processes.

Despite significant progress in the development of the CRP theoretical framework, the defense mechanisms still lack robustness in adversarial scenarios, such as malicious collusion attacks. This has become a key limitation in the practical application of group decision-making systems. Therefore, combining the consensus optimization mechanism of group decision-making with UAV trust management holds great potential for achieving efficient collaborative defense in complex attack scenarios.

## 3. UAV System Architecture

A UAV system consists of four core modules: hardware, software, radio communication links, and application software [28]. The hardware platform includes the flight controller, sensors, actuators, and the UAV body. The flight controller serves as the core unit, coordinating the guidance, control, and navigation systems, while the sensor array collects data such as speed and altitude. The software platform is responsible for UAV trajectory control, the navigation system determines the position, the guidance system directs the UAV to the destination, and the control system ensures precise flight. The communication link is used for data and command transmission, including communication between UAVs and ground stations, inter-UAV communication, and local link communication. Application software is used for task execution, such as trust computation, consistency evaluation, and dynamic adjustments, running on the onboard computer.

**Figure 1:**
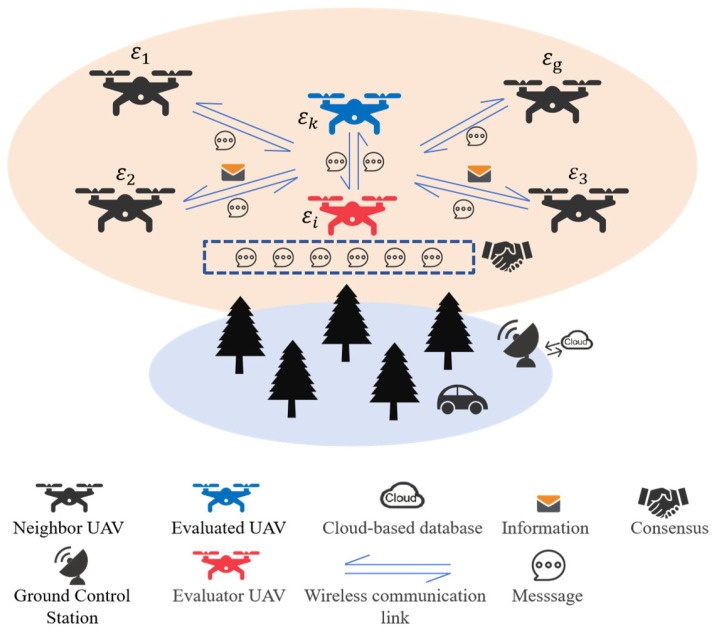
The scenario of GDM-DTM in a low-altitude economy.

The low-altitude economic UAV group is shown in Figure 1, in which all the UAVs simulated in this paper are equipped with onboard computers. The UAVs are divided into three categories: evaluator UAVs, evaluated UAVs, and neighbor UAVs. During data transmission, to ensure security, UAVs first perform an initial trust computation. The evaluator is responsible for conducting consistency trust evaluations based on their opinion and other UAVs’ opinions to select the next-hop UAV. The evaluated UAV is the candidate UAV for information transmission. Neighboring UAVs provide the evaluator with attribute trust values regarding the evaluated UAV to enhance evaluation reliability. Based on the consistency evaluation results, the evaluator transmits the adjusted trust values back to the ground control station, which is then uploaded to the cloud. If a UAV providing services to the ground user is marked as a malicious UAV, the user will receive an alert.

## 4. Proposed GDM-DTM

### 4.1. Overview of the Proposed GDM-DTM

To address the challenges in trust management for UAVs in the low-altitude economy, particularly the limitations in malicious node detection in scenarios without reliance on ground infrastructure and the inadequacy of trust modeling under complex attack scenarios, this paper proposes GDM-DTM, a trust management method based on group decision-making.

**Figure 2:**
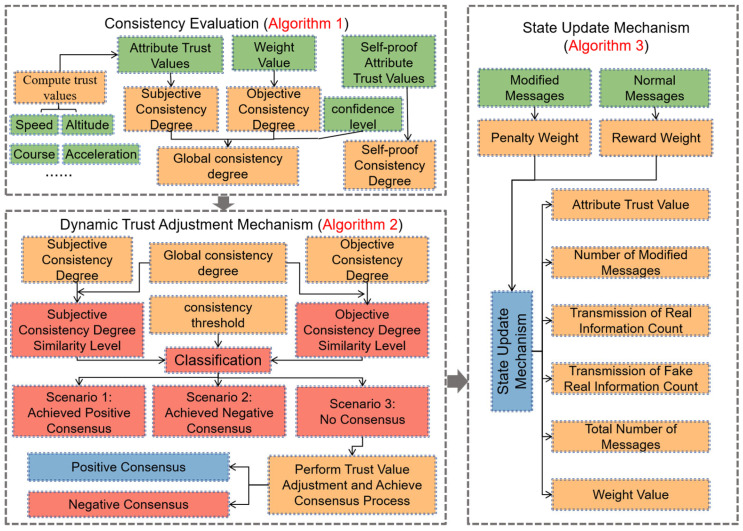
Framework of the proposed GDM-DTM.

GDM-DTM constructs a hierarchical and progressive trust management system by integrating three core components: the Consistency Evaluation Algorithm (Algorithm 1), the Dynamic Trust Adjustment Mechanism (Algorithm 2), and the State Update Mechanism (Algorithm 3), as illustrated in Figure 2. These components are designed to work in a sequential and interdependent manner to support real-time and adaptive trust evaluation in UAV networks under adversarial conditions.

Algorithm 1, called the Consistency Evaluation Algorithm, focuses on measuring the degree of consensus among UAVs from four perspectives—subjective, objective, global, and self-proof consistency—based on multi-dimensional attribute trust values (e.g., speed, altitude, heading, acceleration). This consistency assessment enhances the granularity and credibility of group decision-making.

Algorithm 2, called the Dynamic Trust Adjustment Mechanism, takes the output of the consistency evaluation and compares the consistency degree similarity and the consistency threshold. It classifies the situation into three scenarios: positive consensus, negative consensus, or no consensus, and then triggers corresponding trust value adjustments or additional evaluation rounds. This mechanism improves adaptive responsiveness to inconsistencies and maintains trust system robustness in dynamic environments.

Algorithm 3, called the State Update Mechanism, operates based on the outcomes of UAV information transmission. It applies reward or penalty weights depending on whether the transmitted messages are verified as authentic or tampered. The mechanism updates attribute trust values, weight matrices, message transmission records, and confidence levels accordingly. This enables the system to continuously evolve based on real-time network behavior, ensuring that trust evaluations remain aligned with actual UAV performance.

In summary, Algorithm 1 provides initial consensus estimation, Algorithm 2 ensures adaptive adjustment and resolution of inconsistencies, and Algorithm 3 maintains long-term accuracy by continuously refining trust values and interaction histories. Together, they form a closed-loop system that balances consensus reliability, attack resilience, and real-time adaptability.

### 4.2. Consistency Evaluation Algorithm

To ensure the formation of a robust and multi-perspective consensus evaluation among UAVs, the Consistency Evaluation Algorithm in GDM-DTM is composed of four parts.

Each part focuses on a distinct perspective of trust consensus: the Subjective Consistency Evaluation part is the evaluation perspective of the evaluator UAVs based on multi-dimensional attribute trust; the objective consistency part is the evaluation perspective of aggregating the multi-dimensional attribute trust of neighbor UAVs; the global consistency part integrates subjective and objective evaluations to produce a reasonable global evaluation perspective; and the self-proof consistency part captures the evaluated UAV’s self-proof trust information. The consistency degree of each evaluation perspective is calculated to reflect the trust level of that evaluation perspective, and at the same time, by the consistency similarity degree is calculated between different parts, thereby supporting the dynamic adjustment of trust.

#### 4.2.1. Subjective Consistency Part

- Multi-dimensional Attribute Trust Computation

The trust computation is initialized by evaluating the data transmitted through neighbor UAVs. To address evaluation bias caused by fluctuations in network load, we adopt a trigger condition based on the amount of information k, rather than a fixed time interval. The trust computation process begins when the evaluator receives a set of data from neighbor UAVs. Based on multi-dimensional attribute trust computation, we collect the following series of attributes from the UAV: The evaluator UAV is initialized through trust computation based on multi-dimensional attribute data transmitted from neighbor UAVs. To address evaluation bias caused by fluctuations in network load, we adopt a trigger condition based on the amount of information k, rather than a fixed time interval. The trust computation process begins when the evaluator receives a set of data from neighboring UAVs. Based on multi-dimensional attribute trust computation, we collect the following series of attributes from the UAV: speed (v), defined as the UAV’s current speed; altitude (e), defined as the UAV’s current flight altitude; acceleration (a), defined as the UAV’s current acceleration; and heading (h), which represents the UAV’s heading, among others.

The evaluator UAV $E_{i}$ receives information from the same evaluated UAV $E_{j}$ at different times $\left\{t_{1},t_{2},\ldots ,t_{k}\right\}$ about the same attribute, represented as $M=\{M_{E_{j}}^{t_{1}},M_{E_{j}}^{t_{2}},\ldots ,M_{E_{j}}^{t_{k}}\}\left(\left|M\right|=k\right)$. To compute the attribute trust value for $E_{j}$, the first-order difference $\Delta r_{t}^{\lambda }=\left|\left|r_{t}^{\lambda }\right|-\left|r_{t-1}^{\lambda }\right|\right|$ is calculated for each attribute, where $r_{t}^{\lambda }$ represents the value of attribute λ at time t. The first-order difference represents the numerical change in the attribute value in the information.

Then, based on the statistical distribution characteristics of the attribute values, data screening is performed using the following criterion: if the attribute value $r_{t}^{\lambda }$ satisfies inequality (1), it is considered normal data; otherwise, it is regarded as abnormal data.

(1) $$P_{r}\left\{\left|\Delta r_{t}^{\lambda }-\mu \right|\geq \varepsilon \right\}\leq \frac{\sigma^{2}}{\varepsilon^{2}},$$

where μ and σ represent the mathematical expectation and standard deviation of $\Delta r_{t}^{\lambda }$, respectively, while E determines the magnitude of the occurrence probability.

Finally, the attribute trust value $a_{i,j}^{m}$ is calculated as the ratio of the number of normal data to the total number of received data:

(2) $$a_{i,j}^{m}=\frac{|M|-O^{m}}{\left|M\right|},$$

where m represents the m-th attribute, and $O^{m}$ denotes the number of outlier data for attribute m in the $\left|M\right|$ information. The value of $a_{i,j}^{m}$ lies between 0 and 1.

Based on the GDM framework, in trust management, UAVs make decisions based on their attribute trust values from a set of alternatives. This GDM problem is modeled as a CRP problem. The alternative set is represented as $E=\{E_{1},E_{1},\ldots ,E_{n}\}\left(n\geq 2\right)$, where $E_{n}$ represents the n-th UAV.

Furthermore, the trust matrix $A_{i}$ represents the trust values of the i-th UAV in the attributes of other UAVs, which is used to describe the UAV’s trust in the attributes of other UAVs, as shown in (3):

(3) $$A_{i}={\left[\begin{array}{cccc} a_{i,1}^{1} & a_{i,1}^{2} & \ldots & a_{i,1}^{m}\\ a_{i,2}^{1} & a_{i,2}^{2} & \ldots & a_{i,2}^{m}\\ \vdots & \vdots & \ddots & \vdots \\ a_{i,n}^{1} & a_{i,n}^{2} & \ldots & a_{i,n}^{m} \end{array}\right]}_{n\times m}.$$

In this equation, $A_{i}$ represents the trust values of the i-th UAV in the attributes of other UAVs, where $a_{i,j\;}^{m}$ represents the trust of $E_{i}$ in the m-th attribute of $E_{j}$.

- Weight Calculation

A weight matrix $w_{i}$ based on information feedback is introduced to describe the social relationships of $E_{i}$ as shown in (4).

(4) $$w_{i}={\left[\begin{array}{cccc} w_{i,1}^{1} & w_{i,1}^{2} & \ldots & w_{i,1}^{m}\\ w_{i,2}^{1} & w_{i,2}^{2} & \ldots & w_{i,2}^{m}\\ \vdots & \vdots & \ddots & \vdots \\ w_{i,n}^{1} & w_{i,n}^{2} & \ldots & w_{i,n}^{m} \end{array}\right]}_{n\times m},$$

where $w_{i,n}^{m}$ represents the weight of the m-th attribute of $E_{n}$ at the node of $E_{i}$, corresponding to $a_{i,n}^{m}$.

- Subjective Consistency Degree Calculation

The attribute trust values of the evaluated UAV $E_{k}$, computed by the evaluator UAV $E_{i}$, are aggregated with weights to form the trust value for $E_{k}$, referred to as the subjective consistency degree. This is represented as $SCD_{i,k}$, as shown in (5):

(5) $$SCD_{i,k}=\sum_{j=1}^{m}\;w_{i,k}^{j}a_{i,k}^{j},$$

where m is the number of attributes, and $w_{i,k}^{j}$ represents the weight of the attribute trust value $a_{i,k}^{j}$.

#### 4.2.2. Objective Consistency Part

Based on the attribute trust values of $E_{i}$’s neighbor UAVs $E_{g}$, the weighted aggregation forms the trust value for $E_{k}$, which is referred to as the objective consistency degree. This is denoted as $OCD_{i,k}$, as shown in Equation (6):

(6) $$OCD_{i,k}=\sum_{j=1}^{m}\;w_{i,k}^{j}\left(\sum_{g\in O}^{\left|O\right|}\;\hat{w_{g,k}^{j}}a_{g,k}^{j}\right),$$

where O represents the neighborhood of $E_{i}$ that interacts with $E_{k}$, and $\hat{w_{g,k}^{j}}$ represents the normalized weight based on the set O, given by $\hat{w_{g,k}^{j}}=\frac{w_{g,k}^{j}}{\sum_{t\in O}^{\left|O\right|}w_{t,k}^{j}}$.

#### 4.2.3. Global Consistency Part

The global consistency degree $GCD_{i,k}$ is a comprehensive consistency degree calculated based on $SCD_{i,k}$ and $OCD_{i,k}$, denoted as (7):

(7) $$GCD_{i,k}=\beta SCD_{i,k}+\left(1-\beta \right)OCD_{i,k},$$

where β represents the confidence level of the evaluator UAV $E_{i}$.

#### 4.2.4. Self-Proof Consistency Part

The self-proof consistency degree $SCD_{k}$ represents the trust value provided by $E_{k}$ itself, denoted as (8):

(8) $$SCD_{k}=\sum_{j=1}^{m}w_{i,k}^{j}a_{k}^{j},$$

where $a_{k}^{i}$ represents the self-proven trust level of the j-th attribute. The self-proof consistency degree $SCD_{k}$ is used to determine whether it significantly differs from other consistency degrees. Specifically, if the similarity between $SCD_{k}$ and other consistency degrees does not meet the dynamic consistency threshold, the probability of malicious behavior by $E_{k}$ is high.

#### 4.2.5. Consistency Degree Similarity Calculation

The consistency degree similarity $SCL_{t,k}$ indicates the similarity between $SCD_{k}$ and other consistency degrees, where t∈{i,o,oi}, as shown in (9)–(11):

(9) $$SCL_{i,k}=1-\frac{\left|SCD_{k}-SCD_{i,k}\right|}{\max\left(SCD_{k},SCD_{i,k}\right)},$$

(10) $$SCL_{o,k}=1-\frac{\left|SCD_{k}-OCD_{i,k}\right|}{\max\left(SCD_{k},OCD_{i,k}\right)},$$

(11) $$SCL_{oi,k}=1-\frac{\left|SCD_{k}-GCD_{i,k}\right|}{\max\left(SCD_{k},GCD_{i,k}\right)},$$

where t∈{i,o,oi} is a temporary variable representing the three consistency degrees. The higher the similarity level, the smaller the divergence in attribute trust values. To determine whether the consistency degree similarity is met, a consistency threshold is applied, as shown (12):

(12) $$SCL_{t,k}\in \{SCL_{i,k},SCL_{o,k},SCL_{oi,k}\}\geq \mu ,$$

where μ represents the consistency threshold. When the consistency degree similarity exceeds μ, the self-proof consistency degree can be accepted by the evaluator $E_{i}$. The consensus threshold is given by (13):

(13) $$\mu =\beta \mu_{i}+(1-\beta )\sum_{g\in O}^{\left|O\right|}\;\hat{w_{i,g}}\mu_{g},$$

where $\mu_{i}$ represents the confidence tolerance of $E_{i}$, and $\mu_{g}$ represents the confidence tolerance of $E_{g}$. $\hat{w_{i,g}}$ denotes the normalized weight of $E_{g}$ as viewed from $E_{i}$.

### 4.3. Dynamic Trust Adjustment Mechanism

Based on the consistency evaluation calculation results, a Dynamic Trust Adjustment Mechanism is proposed. This mechanism adjusts the UAV’s attribute trust values according to the relationship between the consistency degree similarity and the consistency threshold. The mechanism mainly includes two scenarios: (1) when the consistency degree similarity between the evaluator UAV and its neighbor UAVs reaches agreement, and (2) when the consistency degree similarity between the evaluator UAV and its neighbor UAVs does not reach agreement.

As outlined in Table 2, the Dynamic Trust Adjustment Mechanism operates as follows. When the subjective and objective consistency degrees are in agreement, the system proceeds based on their unified assessment: if both deem the evaluated UAV benign, the information copy is transmitted to the UAV; conversely, if both classify it as malicious, the UAV is flagged as such, and communication is terminated. In cases where the assessments disagree, the mechanism computes the global consistency degree. Subsequently, the evaluator determines whether an additional round of attribute trust value adjustment is required by comparing the global consistency degree similarity against threshold.

- Case 1: $SCL_{oi,k}\geq \mu$;

This case indicates that the evaluator UAV $E_{i}$ has strong trust in the evaluated UAV $E_{k}$. To avoid overconfidence from $E_{i}$ leading to information being transmitted to malicious UAVs, $E_{i}$ adjusts the trust value of the j-th attribute with the greatest divergence between itself and its neighbors regarding $E_{k}$ (denoted as $D_{ok}^{j}$) to align with the attribute trust values of its neighbors. $D_{ok}^{j}$ is calculated as (14):

(14) $$D_{ok}^{j}=\sum_{g\in O}^{\left|O\right|}\;\hat{w_{g,k}^{j}}(a_{g,k}^{j}-a_{k}^{j}),$$

where $\hat{w_{g,k}^{j}}$ represents the normalized weight of the j-th attribute trust value, and $a_{k}^{j}$ represents the self-proven trust value of the j-th attribute. The attribute corresponding to the maximum $D_{ok}^{j}$ is found and adjusted according to the following rule:

(15) $$a_{i,k}^{j*}=\beta a_{i,k}^{j}+\left(1-\beta \right)\sum_{g\in O}^{\left|O\right|}\;\hat{w_{g,k}^{j}}a_{g,k}^{j},$$

where $a_{i,k}^{j*}$ represents the adjusted trust value of the j-th attribute for $E_{i}$. The new global consistency degree similarity $SCL_{oi,k}^{\prime }$ is then recalculated. If $SCL_{oi,k}^{\prime }\geq \mu$, $E_{i}$ sends the marked information to $E_{k}$; if $SCL_{oi,k}^{\prime }<\mu$, $E_{k}$ is considered a malicious UAV.

- Case 2: $SCL_{oi,k}<\mu$;

This case indicates that $E_{i}$ has a positive evaluation of $E_{k}$, but does not fully trust $E_{k}$. To mitigate the risk of collusion attacks between $E_{k}$ and its neighbors, $E_{i}$ sends a marking message if its confidence exceeds 0.5 and it can bear the responsibility for potential information transmission failure. Otherwise, $E_{k}$ is recognized as a malicious UAV, and no information is transmitted.

- Case 3: $SCL_{oi,k}\geq \mu$;

This case indicates that $E_{i}$ does not fully trust $E_{k}$, but its neighbors have a positive evaluation of $E_{k}$. To overcome $E_{i}$’s bias, $E_{i}$ integrates the attribute trust values of its neighbors. The attribute trust value with the greatest divergence between $E_{i}$ and $E_{k}$ for the j-th attribute (denoted as $D_{ik}^{j}$) is found using the following rule:

(16) $$D_{ik}^{j}=\left|a_{i,k}^{j}-a_{k}^{j}\right|.$$

The corresponding attribute trust value is then adjusted according to Equation (15). The new global consistency degree similarity $SCL_{oi,k}^{\prime }$ is recalculated. If $SCL_{oi,k}^{\prime }\geq \mu$, the marking message is sent; otherwise, $E_{k}$ is considered a malicious UAV.

- Case 4: $SCL_{oi,k}<\mu$;

If the confidence level of the neighbors exceeds 0.5, $E_{i}$ generates a marking message for $E_{k}$. Otherwise, $E_{k}$ is classified as a malicious UAV, and $E_{i}$ will not transmit any information to $E_{k}$.

### 4.4. State Update Mechanism

Before information transmission, the expected attribute trust values are pre-computed to facilitate updates when the information reaches its destination. The system dynamically updates the attribute trust value weights based on the amount of information transmitted. A message matrix is then introduced to update the UAV’s weights (e.g., $w_{i,n}$), as shown (17):

(17) $$D_{i}={\left[\begin{array}{c} \left\{d_{i,1}^{s},d_{i,1}^{t}\},\{d_{i,2}^{s},d_{i,2}^{t}\},\ldots ,\{d_{i,n}^{s},d_{i,n}^{t}\right\} \end{array}\right]}_{1\times n},$$

In this equation, $d_{i,n}^{s}$ represents the number of untampered messages received by node $E_{i}$ through $E_{n}$, and $d_{i,n}^{t}$ represents the total number of messages transmitted through $E_{n}$. Specifically, each message contains a list representing the sequence of nodes it has passed through. If the message successfully reaches the destination node and is untampered with, the corresponding value in the message matrix and the weights of the traversed nodes are updated as $d_{i,g}^{s*}=d_{i,g}^{s}+1$. Then, the weight $w_{i,g}$ is updated as $w_{i,g}=\frac{d_{i,g}^{s*}}{d_{i,g}^{t}}$, where $w_{i,g}$ represents the weight of $E_{g}$ from the perspective of $E_{i}$.

For Case 1, when the attribute trust values of the evaluator are closer to those of its neighbors, the following formula applies:

(18) $$a_{i,k}^{j*}=\beta a_{i,k}^{j}+(1-\beta )\sum_{g\in O}^{\left|O\right|}\;\hat{w_{g,k}^{j}}a_{g,k}^{j}.$$

For Case 2, no information transmission occurs, and no update to the attribute trust values is made.

For Cases 3 and 4, the expected attribute trust value calculation is divided into two cases based on whether a marking message is sent. If no message is sent, the formula is as follows:

(19) $$a_{i,k}^{j*}=max\{\beta a_{i,k}^{j}+(1-\beta )\frac{\left|\sum_{g\in O}^{\left|O\right|}\;\hat{w_{g,k}^{j}}a_{g,k}^{j}-a_{i,k}^{j}\right|}{max\{\sum_{g\in O}^{\left|O\right|}\;\hat{w_{g,k}^{j}}a_{g,k}^{j},a_{i,k}^{j}\}},a_{i,k}^{j}\},$$

where $\left|\sum_{g\in O}^{\left|O\right|}\;\hat{w_{g,k}^{j}}a_{g,k}^{j}-a_{i,k}^{j}\right|$ quantifies the difference between objective consensus and subjective consensus. This difference metric ensures that the global consensus improves the accuracy of benign UAV recognition and encourages trust evaluations to be more consistent across all parties. When a marking message is sent, the neighbors $E_{g}$ provide critical attribute trust values to encourage the evaluator $E_{i}$ to transmit information. Therefore, the update rule is related to $E_{g}$ as follows:

(20) $$a_{i,k}^{j*}=\beta a_{i,k}^{j}+(1-\beta )\sum_{g\in O}^{\left|O\right|}\;\frac{a_{i,g}^{j}+a_{i,k}^{j}}{2m}.$$

When the information reaches the final destination node, the node checks the authenticity of the received information, generates a broadcast list, and notifies the UAVs to update their attribute trust values, weight matrices $w_{i}$, and information record matrices.

If the transmitted information has been tampered with, the attribute trust value of $E_{i}$ for $E_{k}$ will be reduced according to the following formula:

(21) $$\bar{a_{i,k}^{j}}=a_{i,k}^{j}\cdot e^{-\gamma \cdot \left|a_{i,k}^{j*}-a_{i,k}^{j^{\prime }}\right|},$$

where $a_{i,k}^{j*}$ represents the expected attribute trust value of $E_{i}$ for $E_{k}$, and $a_{i,k}^{j^{\prime }}$ represents the attribute trust value before the corresponding information transmission. $a_{i,k}^{j}$ is the current attribute trust value. γ controls the strength of the adjustment, increasing sensitivity to repeated tampering. The information matrix records are then adjusted to reduce the weight of $E_{k}$ and its guarantor $E_{g}$. The adjustment formula is as follows:

(22) $$d_{i,j}^{t*}=d_{i,j}^{t}\times \gamma ,j\in \{k,g\},$$

where $d_{i,j}^{t*}$ refers to the updated total number of information transmissions from $E_{i}$ to $E_{j}\in \{E_{k},E_{g}\}$. Then, the weight of $E_{k}$ on $E_{i}$ will decrease according to $w_{i,g}=\frac{d_{i,g}^{s}}{d_{i,g}^{t}}$.

If the information has been verified, the attribute trust value is increased based on the current and expected attribute trust value increments, as follows:

(23) $$\bar{a_{i,k}^{j}}=a_{i,k}^{j}+\left(1-a_{i,k}^{j}\right)\cdot \left(1-e^{-\gamma \cdot \left|a_{i,k}^{j*}-a_{i,k}^{j^{\prime }}\right|}\right),$$

where $\left|a_{i,k}^{j*}-a_{i,k}^{j^{\prime }}\right|$ represents the increment in the expected attribute trust value. The number of untampered messages $d_{i,k}^{s}$ and the total transmitted messages $d_{i,k}^{t}$ will increase as $d_{i,k}^{s*}=d_{i,k}^{s}+1,d_{i,k}^{t*}=d_{i,k}^{t}+1$. Additionally, the weights of the evaluated UAV and its neighbors are updated. The result of data transmission affects the evaluator’s confidence level, and the update formula is as follows:

(24) $$\beta =1-\frac{F}{T+F},$$

where T represents the number of truthful messages sent, and F represents the number of tampered messages.

## 5. Performance Evaluation

### 5.1. Simulation Setup

The experimental simulations were conducted using the Opportunistic Network Environment (ONE) Simulator version 1.6.0 [29]. The UAV flight paths were modeled based on a digital representation of downtown Helsinki. To ensure the relevance and validity of the simulation, key parameters were selected based on established research practices and validated reference models in the literature [30,31,32,33]. Specifically, the simulation duration was set to 5000 s, providing adequate time for observing UAV behavior patterns and trust dynamics. A total of 120 UAVs were randomly deployed across the simulation map to simulate realistic node density and network interactions. The main simulation parameters are shown in Table 3.

UAV mobility speeds are uniformly distributed between 3 m/s and 15 m/s, reflecting typical operational speeds in low-altitude urban environments. The UAV communication range of 300 m was chosen based on common short-range wireless communication standards used in existing UAV network simulations. The bandwidth was set to 5 Mbit/s, sufficient for the transmission of typical data payloads expected in UAV communications. Additionally, each UAV was equipped with a storage buffer of 2 GB to manage data during transit effectively.

In the experiment, UAVs were categorized into benign and malicious UAVs, with each group following distinct movement patterns based on their role. The benign UAVs move according to their task types and the specified target locations, performing their missions with predefined paths. On the other hand, malicious UAVs behave unpredictably, following random routes, and intentionally tampering with their attributes such as speed and heading. Malicious UAVs generate and broadcast false information to neighboring UAVs, contributing to deception in the network. Meanwhile, benign UAVs generate 2 MB sized information, which is accurately transmitted as part of the network’s data flow.

To evaluate the performance of the proposed GDM-DTM, we conducted comprehensive experiments focusing on three key aspects: (1) UAV trust value calculation evaluation, (2) trust management system performance analysis, and (3) comparative benchmarking against existing models. Specifically, the trust value calculation evaluation analysis will track the changes in trust values for both benign and malicious UAVs over time to verify the stability and accuracy of the multi-dimensional attribute trust computation model and Dynamic Trust Adjustment Mechanism. System performance was evaluated using accuracy, precision, recall, and F-score metrics under different malicious ratios and message densities to assess the system’s ability to detect malicious UAVs. Furthermore, the proposed method was compared with D2MIF, FedTrust, and B5G (SVM) to comprehensively evaluate the advantages of the GDM-DTM method in terms of malicious node detection accuracy, model robustness, dynamic environmental adaptability, and overall performance.

### 5.2. UAV Trust Value Calculation Evaluation Analysis

**Figure 3:**
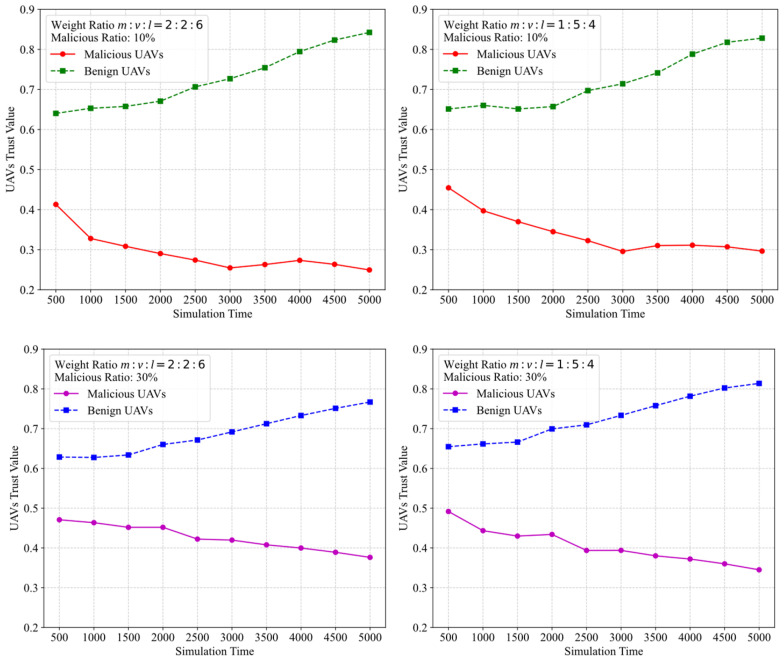
Trust evaluation over simulation time.

Figure 3 shows the impact of different weight configurations on the UAV trust value changes over time in scenarios where the malicious node ratios are 10% and 30%. Specifically, the experiment examines three key attributes: speed (v), location coordinates (l), and message volume (m), with two different weight combinations: m:v:l = 2:2:6 (left) and m:v:l = 1:5:4 (right).

The experimental results indicate that although different weight combinations affect the overall trust level, the trust mean for benign UAVs increases over time across all configurations, while the trust mean for malicious UAVs gradually decreases. As the malicious ratio increases, the rate of change of the trust value decreases. The core mechanisms behind these findings are as follows: (1) The Dynamic Trust Adjustment Mechanism adjusts trust values based on the results of information transmission, enhancing the trust in benign nodes and suppressing malicious nodes. (2) Multi-level consistency evaluation (subjective, objective, global, and self-proof consistency) comprehensively identifies abnormal attributes, such as the divergence between global consensus and self-proof trust, triggering dynamic trust adjustments and improving detection sensitivity. (3) The dynamic update strategy for the weight matrix and confidence level optimizes the reliability and anti-interference capability of group decision-making. (4) The accumulation of information over time in the simulation provides data support for multi-dimensional attribute statistical analysis, effectively weakening the short-term camouflage effects of malicious nodes. In conclusion, the experimental results validate the system’s effectiveness in distinguishing benign and malicious UAVs. The system ensures trust value reliability with good robustness across different malicious node ratios and weight configurations.

### 5.3. UAV Trust Management System Performance Analysis

#### 5.3.1. Attack Model

The message tampering and fake information generation attack model (MTGI) was adopted. Malicious UAVs tamper with the data upon receiving messages from benign UAVs and perform transmission. At the same time, malicious UAVs randomly generate fake information to interfere with other benign UAVs. Malicious UAVs recognize each other, while benign UAVs are unaware of this.

#### 5.3.2. Performance Evaluation Under MTGI

We present definitions below:

**(i) True Positive (TP)**: The device is predicted to be malicious, and it is actually malicious.

**(ii) True Negative (TN)**: The device is predicted to be benign, and it is actually benign.

**(iii) False Negative (FN)**: The device is predicted to be benign, but it is actually malicious.

**(iv) False Positive (FP)**: The device is predicted to be malicious, but it is actually benign.

- **Accuracy:** Represents the percentage of correctly identified results.

$$Accuracy=\frac{TP+TN}{TP+TN+FP+FN}$$

- **Precision:** Represents the proportion of predicted malicious nodes that are actually malicious.

$$Precision=\frac{TP}{TP+FP}$$

- **Recall**: Represents the proportion of actual malicious nodes that are correctly identified.

$$Recall=\frac{TP}{TP+FN}$$

- **F-score:** A performance measure calculated from precision and recall.

$$F_{\beta }=\left(1+\beta^{2}\right)\cdot \frac{\mathrm{Precision}\cdot \mathrm{Recall}}{\beta^{2}\cdot Precision+\mathrm{Recall}}$$

Accuracy, precision, and recall are highly correlated with the ratio of benign to malicious UAVs. In cases of low malicious ratios, although higher accuracy and recall can be achieved, the precision is lower. This is an undesirable result for systems requiring high security or robustness. Although these three metrics focus on different aspects of identification, they fail to find a balance between them. Therefore, to balance precision and recall, the F-score (F value) metric is used.

- **Accuracy Performance**

**Figure 4:**
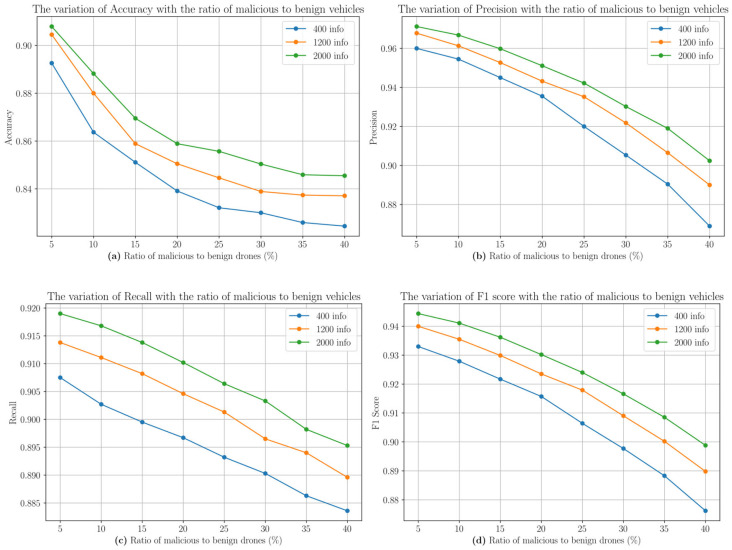
The performance of GDM-DTM under MTGI in terms of (**a**) accuracy, (**b**) precision, (**c**) recall, and (**d**) F-score.

As shown in Figure 4a, the system’s accuracy gradually decreases as the ratio of malicious UAVs (ξ) increases. When the number of messages is 2000, the accuracy reaches 90.79% at ξ=5% and remains at 84.55% when ξ=40%. Notably, the amount of information has a significant impact on system performance: when ξ=20%, increasing the number of messages from 400 to 2000 results in a 1.98% increase in accuracy (83.91% to 85.89%). This phenomenon stems from the dual effects of the Dynamic Trust Adjustment Mechanism: on one hand, the weight normalization strategy weakens the impact of coordinated attacks by malicious nodes on a single attribute; on the other hand, the confidence update model introduced in Equation (24) incorporates the feedback on information authenticity into the evaluation system, effectively suppressing the self-reinforcing effect of collusive networks. The experimental results show that the system maintains 83% detection accuracy even in high malicious ratio scenarios (ξ=30%), validating the environmental adaptability of the group decision-making mechanism.

- **Precision**

As shown in Figure 4b, precision decreases non-linearly with an increasing malicious ratio. For the scenario with 2000 messages, as ξ increases from 5% to 40%, precision drops from 97.12% to 90.24%. This relatively smooth decline stems from the filtering mechanism of the self-proof consensus part: when the self-proof trust value generated by malicious nodes through Equation (8) deviates from other consistency parts beyond the dynamic threshold μ (Equation (13)), the system calls the Dynamic Trust Adjustment Mechanism. For example, if a malicious node falsifies a 10° deviation in heading, the objective consensus part detects this anomaly through multi-source verification data from neighboring nodes, triggering trust value weight correction towards the objective consensus direction (Equation (20)).

- **Recall**

As shown in Figure 4c, recall decreases gradually as the malicious UAV ratio increases. For the scenario with 2000 messages, recall decreases from 91.90% to 89.53% as ξ increases from 5% to 40%, verifying the system’s robustness under high malicious density. This robustness is primarily due to the combined effects of the multi-dimensional trust evaluation mechanism: cross-validation of multiple attributes such as speed and altitude effectively identifies short-term camouflage behaviors; Self-Proof Consistency Evaluation (Equation (7)) dynamically detects deviations between self-proof trust values and global consensus, such as heading differences triggering adjustments (Equation (15)), reducing the risk of false negatives. The synergy of the multi-level consensus mechanism and dynamic feedback ensures that the system maintains a high recall rate in complex attack scenarios.

- **F-score**

The F-score is the harmonic mean of precision and recall and is used to assess the overall classification performance of the model. As shown in the experimental data in Figure 4d, the F-score decreases gradually as the malicious UAV ratio (ξ) increases, but the decline is relatively smooth. Under different malicious ratios (5–40%) and message volumes (400–2000 messages), the F-score remains at a high level (87.62–94.44%), indicating that the GDM-DTM system maintains good stability even at higher malicious node ratios. This is primarily due to the collaborative effects of multi-dimensional trust computation and dynamic adjustment mechanisms; self-proof consistency quickly identifies abnormal behaviors by comparing node self-proof data with other consensus (Equations (9)–(11)), while dynamic weight updates (Equations (17)–(20)) continuously optimize trust evaluation based on information exchange.

#### 5.3.3. Comparison of GDM-DTM with Other Models

**Figure 5:**
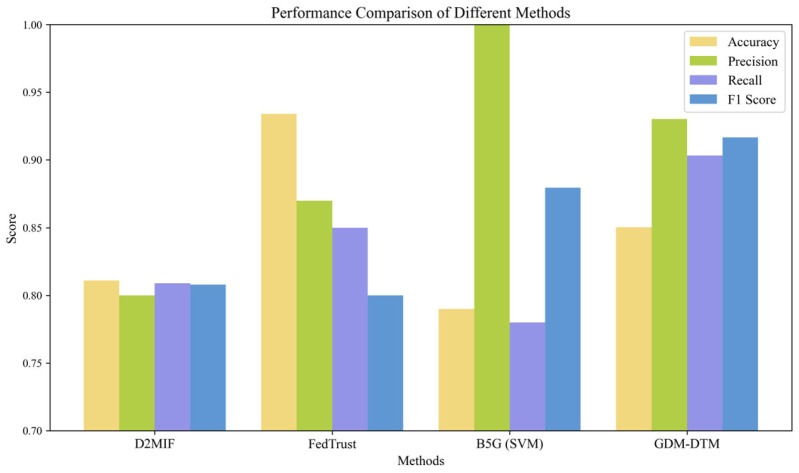
Performance comparison of GDM-DTM, D2MIF, FedTrust, and B5G (SVM).

Based on the aforementioned experiments, we compared the performance of GDM-DTM, D2MIF [34], FedTrust [35], and B5G (SVM) [36] in a scenario where the malicious UAV ratio is 30% and 2000 messages are transmitted to demonstrate their performance. Figure 5 shows the accuracy, precision, recall, and F-score for D2MIF, FedTrust, and B5G (SVM) in the MTGI scenario for malicious node detection models. In terms of accuracy, FedTrust (93.4%) and GDM-DTM (85.04%) perform best, but GDM-DTM leads in recall (90.33%) and F-score (91.66%), reflecting its advantages in comprehensive detection and dynamic balance. D2MIF, based on isolation forests and reinforcement learning, has balanced accuracy (81.1%) and recall (80.9%) but a lower F-score (80.8%), likely due to insufficient dynamic threshold adjustment leading to false positives. B5G (SVM) achieves 100% precision but only a 78% recall, indicating that its strict filtering strategy may miss detecting real malicious nodes. FedTrust, relying on deep federated learning, has the highest accuracy but a low F-score (80%), reflecting its weaker adaptability to imbalanced data. In contrast, GDM-DTM maintains 85.04% accuracy and 91.66% F-score under high malicious node ratios, leveraging multi-dimensional trust evaluation and group decision-making mechanisms, effectively suppressing collusive attacks and enhancing robustness, offering superior overall performance.

## 6. Conclusions and Future Directions

This study investigates the trust management problem in low-altitude economy UAV networks and proposes a consistency evaluation trust management method based on group decision-making. By integrating group decision-making and multi-dimensional attribute data, this method provides a solution for malicious node detection in UAV networks. The experimental results show that the proposed method demonstrates high accuracy and robustness in detecting malicious UAVs and propagating false information. Although there are certain limitations in terms of computational complexity and adaptability to real-world scenarios, future research could optimize algorithms, expand application scenarios, and integrate technologies such as blockchain to further enhance the system’s real-time capabilities and security. This research provides new insights into UAV trust system network and promotes the safe development of the low-altitude economy.

## Figures and Tables

**Table 1:** Comparison of functionality.

	Independent of Ground Infrastructure	Multi-Dimensional Trust Modeling	Resistance to Collusion Attacks	Dynamic Threshold/Weight Adjustment	Group Decision-Making Mechanism	Self-Proof Consistency Verification
GDM-DTM (ours)	✓	✓	✓	✓	✓	✓
LTME [20]	✗	✗	✗	✓	✗	✗
TDLS-FANET [21]	✗	✓	✗	✗	✗	✗
UTCD [22]	✗	✗	✓	✗	✗	✓
Rep-Raft-Poa [23]	✗	✗	✓	✗	✗	✗
GALTrust [24]	✗	✓	✓	✓	✗	✗
TMIoDT [25]	✗	✗	✗	✗	✗	✗

**Table 2:** Process of the Dynamic Trust Adjustment Mechanism.

Scenario	Condition	Operation
Agreement between subjective and objective (judged as benign)	$SCL_{i,k},\;SCL_{o,k}\geq \mu$	$\mathrm{Send}\;\mathrm{the}\;\mathrm{information}\;\mathrm{copy}\;\mathrm{to}\;E_{k}$
Agreement between subjective and objective (judged as malicious)	$SCL_{i,k},\;SCL_{o,k}<\mu$	Mark as malicious node, block communication
Conflict between subjective and objective (subjective judged as benign)	$SCL_{i,k}\geq \mu ,\;SCL_{o,k}<\mu$	$\mathrm{Compare}\;SCL_{oi,k}\;$and μ, divide into Case 1 and Case 2
Conflict between subjective and objective (subjective judged as malicious)	$SCL_{i,k}<\mu ,\;SCL_{o,k}\geq \mu$	$\mathrm{Compare}\;SCL_{oi,k}\;$and μ, divide into Case 3 and Case 4

**Table 3:** Simulation parameters.

Parameter	Value
Simulation time	5000 s
Number of drones	120
Speed distribution	[3~15] m/s
Storage buffer	2 G
Bandwidth transfer speed	5 Mbit/s
Communication range	300 m

## Data Availability

Data are contained within the article.
